# Supplementary material for: Chromoblastomycosis: A case series from Eastern China
Source: PLoS Negl Trop Dis. 2022 Sep 26;16(9):e0010800. doi: 10.1371/journal.pntd.0010800 (PMC9536625; doi:10.1371/journal.pntd.0010800)
Supplement: Attachment — Submitted filename: PNTD-D-22-00708_reviewer.pdf [file pntd.0010800.s002.pdf]

PLOS Neglected Tropical Diseases

Chromoblastomycosis: A case series from eastern China

--Manuscript Draft--

**Manuscript Number:**	PNTD-D-22-00708
**Full Title:**	Chromoblastomycosis: A case series from eastern China
**Short Title:**	Chromoblastomycosis
**Article Type:**	Research Article
**Keywords:**	Chromoblastomycosis; Fonsecaea monophora,Zhejiang,Epidemiology
**Abstract:**	Chromoblastomycosis (CBM), caused by a group of dematiaceous fungi, is a chronic fungal disease mostly affected the skin and subcutaneous tissue. Fonsecaea monophora is one of the most common pathogen of CBM in China. Most of formal cases have been reported from southern China,though the infection is not uncommon in eastern China, a very few case series areavailable from eastern china. To describe the clinical aspects of CBM,we report a series of 11 cases between 2018 and 2021 at a single medical center in eastern china. Patients were predominately male (4.5:1). The disease duration ranged from 3 months to 20 years. Plaque type was the most common clinical manifestations. ALL isolates were identified by morphology and sequencing the ITS regions of ribosomal DNA, 10 were F. monophora and 1 were Cladophialophora carrionii .
**Additional Information:**	
**Question**	**Response**
**Financial Disclosure**<br><br>Enter a financial disclosure statement that describes the sources of funding for the work included in this submission. Review the submission guidelines for detailed requirements. View published research articles from *PLOS NTDs* for specific examples.<br><br>This statement is required for submission and **will appear in the published article** if the submission is accepted. Please make sure it is accurate.	This work was kindly supported by the Hangzhou Science and Technology Bureau, China (Grant No. 202004A17).

Powered by Editorial Manager® and ProduXion Manager® from Aries Systems Corporation

**Unfunded studies**<br><br>Enter: *The author(s) received no specific funding for this work.*<br><br>**Funded studies**<br><br>Enter a statement with the following details:<br>• Initials of the authors who received each award<br>• Grant numbers awarded to each author<br>• The full name of each funder<br>• URL of each funder website<br>• Did the sponsors or funders play any role in the study design, data collection and analysis, decision to publish, or preparation of the manuscript?<br>• **NO** - Include this sentence at the end of your statement: *The funders had no role in study design, data collection and analysis, decision to publish, or preparation of the manuscript.*<br>• **YES** - Specify the role(s) played.<br><br>* typeset	
**Competing Interests**<br><br>Use the instructions below to enter a competing interest statement for this submission. On behalf of all authors, disclose any competing interests that could be perceived to bias this work—acknowledging all financial support and any other relevant financial or non-financial competing interests.<br><br>This statement **will appear in the published article** if the submission is accepted. Please make sure it is accurate. View published research articles from *PLOS NTDs* for specific examples.	The authors have declared that no competing interests exist.

**NO authors have competing interests**<br><br>Enter: *The authors have declared that no competing interests exist.*<br><br>**Authors with competing interests**<br><br>Enter competing interest details beginning with this statement:<br><br>*I have read the journal's policy and the authors of this manuscript have the following competing interests: [insert competing interests here]*<br><br>* typeset<br><br>This statement is required for submission and **will appear in the published article** if the submission is accepted. Please make sure it is accurate and that any funding sources listed in your Funding Information later in the submission form are also declared in your Financial Disclosure statement.	
**Data Availability**<br><br>Authors are required to make all data underlying the findings described fully available, without restriction, and from the time of publication. PLOS allows rare exceptions to address legal and ethical concerns. See the PLOS Data Policy and FAQ for detailed information.	Yes - all data are fully available without restriction

Powered by Editorial Manager® and ProduXion Manager® from Aries Systems Corporation

A Data Availability Statement describing where the data can be found is required at submission. Your answers to this question constitute the Data Availability Statement and **will be published in the article**, if accepted.<br><br>**Important:** Stating 'data available on request from the author' is not sufficient. If your data are only available upon request, select 'No' for the first question and explain your exceptional situation in the text box.<br><br>Do the authors confirm that all data underlying the findings described in their manuscript are fully available without restriction?	
**Describe where the data may be found in full sentences. If you are copying our sample text, replace any instances of XXX with the appropriate details.**<br><br>• If the data are **held or will be held in a public repository**, include URLs, accession numbers or DOIs. If this information will only be available after acceptance, indicate this by ticking the box below. For example: *All XXX files are available from the XXX database (accession number(s) XXX, XXX.*).<br>• If the data are all contained **within the manuscript and/or Supporting Information files**, enter the following: *All relevant data are within the manuscript and its Supporting Information files.*<br>• If neither of these applies but you are able to provide **details of access elsewhere**, with or without limitations, please do so. For example:<br><br>*Data cannot be shared publicly because*	All relevant data are within the manuscript and its Supporting Information files.

of [XXX]. Data are available from the XXX Institutional Data Access / Ethics Committee (contact via XXX) for researchers who meet the criteria for access to confidential data.<br><br>*The data underlying the results presented in the study are available from (include the name of the third party and contact information or URL).*<br>• This text is appropriate if the data are owned by a third party and authors do not have permission to share the data.<br><br>* typeset	
Additional data availability information:	

Powered by Editorial Manager® and ProduXion Manager® from Aries Systems Corporation

Manuscript

Click here to access/download;Manuscript;renamed_d414f.docx

Title: Chromoblastomycosis : A case series from eastern China

Running Title: Chromoblastomycosis

Authors: Sujun Liu[1],Huilin Zhi[1],Hong Shen[1],Wenwen Lv[1],Bo Sang[1],Qiuping Li[1],Yan Zhong[1],Zehu Liu[1],Xiujiao Xia[1*]

[*]Correspondence to:Prof Xiujiao Xia,Department of Dermatology, Hangzhou Third People's Hospital, Affiliated Hangzhou Dermatology Hospital, Zhejiang University School of Medicine, West Lake Rd 38, Hangzhou 310009, PR China

804534095@qq.com ORCID number:0000-0002-3790-8026

Affiliations:[1]Department of Dermatology, Hangzhou Third People's Hospital, Affiliated Hangzhou Dermatology Hospital, Zhejiang University School of Medicine, West Lake Rd 38, Hangzhou 310009, PR China

Acknowledgments: This work was kindly supported by the Hangzhou Science and Technology Bureau, China (Grant No. 202004A17).

Conflict of Interest: No author has a conflict of interest.

Ethics committee approval:Written and informed consent was obtained from patients. The study was approved by the Medical Ethics Committee at the Hangzhou Third People's Hospital (No.2020KA005)

Author contributions: SL Lliu wrote the manuscript, collected figures, and reviewed the literature. HL Zhi,WW Lv,B Sang,QP Li and Y Zhong performed mycological examination.ZH Liu and H Shen executed treatment protocols.

Data availability statement:All data generated or analysed during this study are included in this published article.

Abstract

Chromoblastomycosis (CBM), caused by a group of dematiaceous fungi, is a chronic fungal disease mostly affected the skin and subcutaneous tissue. *Fonsecaea monophora* is one of the most common pathogen of CBM in China. Most of formal cases have been reported from southern China,though the infection is not uncommon in eastern China, a very few case series areavailable from eastern china. To describe the clinical aspects of CBM,we report a series of 11 cases between 2018 and 2021 at a single medical center in eastern china. Patients were predominately male (4.5:1). The disease duration ranged from 3 months to 20 years. Plaque type was the most common clinical manifestations. ALL isolates were identified by morphology and sequencing the ITS regions of ribosomal DNA, 10 were *F. monophora* and 1 were *Cladophialophora carrionii*.

Key words: Chromoblastomycosis, *Fonsecaea monophora*,Zhejiang,Epidemiology

Introduction

Chromoblastomycosis (CBM) is an implantation mycosis, consisting of chronic cutaneous and subcutaneous lesions that develop at the site of previous transcutaneous trauma.[1] CBM is strongly associated with agricultural activities and the lack of protection, which further underscores the occupational nature of this disease. [2] It can lead to chronic persistent infections and may cause incapacity for labor in some severe clinical forms. Though known for 100 years,CBM still poses a therapeutic challenge to clinicians due to its recalcitrant nature and common relapse after treatment.[3] The species of the genus Fonsecaea are the most common etiological agent of CBM, and comprises four associated species: *F. pedrosoi*, *F.monophora*, *F. nubica* and *F. pugnacious*.[4-7] *Cladophialophora carrionii* was the most common causative agent in the north of the Mainland China, and *F. monophora* and *F. pedrosoi* were the most common agents in the southern part of the Mainland China.[8] A comprehensive evaluation with molecular sequencing data showed that *F. monphora* was the most

prevalent pathogen of CBM and *F. pedrosoi* has not been isolated from chromoblastomycosis patients in Guangdong, Southern China.[9] In order to evaluate the clinical characteristics of CBM in Zhejiang,eastern China,we reviewed 11 cases at Department of Dermatology of Hangzhou Third people·s Hospital between 2018 and 2021 and analyzed their clinical features, mycological findings, pathogens, comorbidities,treatments, and outcomes.

Methods

A prospective descriptive study of patients with clinically suspected CBM or another importmant chronic dermatomycosis was performed at the medical mycology laboratory in the Hangzhou Third people·s Hospital. Patients were recruited between January 2018 and December 2021 and during consultation campaigns conducted in Zhejiang Province. Diagnosis of these cases was made by clinical observation, mycological examination and histopathology of biopsies collected from scrapings of skin lesions.Age, sex, and occupation,as well as clinical data, such as comorbidities, traumatic history, duration of the lesions, clinical type of skin lesions were recorded. Diagnosis was based on the definitions described and proposed by Carrión in 1950 in which the lesions are divided into five different types (verrucous, nodular, plaque, cicatricial and tumoral).[10] This study was approved by the ethics committee of our hospital and study participants were informed about the study procedures and written informed consent was obtained.

Sample collectiont Sterilized lancet was used for scraping the surface of the lesions, principally the more highly pigmented areas known as black dots,and pus samples were collected by squeezing the skin lesions. Skin biopsies were performed in all cases, and portions of the specimens were fixed in 10% neutral buffered formalin, embedded in paraffin, sectioned and stained with haematoxylin & eosin (H&E), periodic acid-Schiff (PAS), another fresh portions were directly inoculated onto Sabouraud dextrose agar (SDA).

Mycological examination KOH preparation of scales and pus obtained from the lesions was examined under a microscope for muriform bodies or septate dematiaceous hyphae . A separate scales or pus sample was used for fungal culture. Portions of both skin biopsies and skin debris were inoculated onto culture media attempting to recover the etiologic agent. Primary isolation of the fungus was performed on agar slants of SDA containing chloramphenicol (CMP, 0.125 g/l) and incubated at 25 °C for two weeks. The isolates were identified by slide-culture microscopy. The final identification of isolated agent was confirmed by sequencing of ITS 1 and ITS 4 region of rDNA and compared with sequences deposited in GenBank by Blast program.

Results

The clinical data of nine men and two women are summarized in Table 1. The age ranged from 39 to 83 years (mean of 62.9 years). There were six farmers,three carpenters,one gardener,and one retired teacher who planted a variety of flowers at home. The duration of CBM ranged from 3 months to 20 years (mean of 6.5 years), but most of the patients suffered from the infection for more than 1 year (75%). Six patients (54.5%) either could not recall or gave a negative history of trauma prior to their manifestations. Among five cases with history of trauma,three cases caused by wooden spike prick,two cases caused by insect bite. Diabetes was the most common comorbidity. Three patients had (27.2%) had diabetes,two patients had chronic kidney disease,one patient had diffuse pulmonary interstitial fibrosis with hypertension and one patient had heart disease. The lesions were localized to 1 site in 63.6% of cases (7/11). The most common site afflicted was upper limb (7 cases) followed by lower limb (2 cases),buttocks (1 case), and trunk (1 case). The most common clinical variety described was plaque (63.6%,7/11) followed by verrucous (18.2%,2/11), nodular and cicatricial (9.1% each) (Fig.1A-E).

Before consultation campaigns,all cases had been evaluated at another clinics, eight cases were misdiagnosed as other diseases,of them, sporotrichosis and eczema were the most common diagnosis,followed by lupus vulgaris and neurodermatitis.

All cases of direct mycological examination were positive,skin scrapings or pus treated with 10% KOH revealed muriform cells with or without germinated hyphae (Fig.2A-B). 5 cases were histopathologically proven with the characteristic brownish thick-walled sclerotic bodies being demonstrable in all the cases either within or outside the giant cells (Fig.2C). Pathogens were isolated from eleven cases. Ten pathogens were identified as *C.carrionii* (Fig.2D)and one as *F. pedrosoi* (Fig.2E-F) based on their morphologica characters.Based on the sequences of the ITS1-5.8S-ITS2 region of rDNA, nine *F. pedrosoi* isolates reidentified as *F.monophora*.

Of the eleven cases, nine cases received systemic treatment in our hospital , cases 1 gave up treatment due to the high cost of treatment and case 10 returned to the local area for treatment. Eight cases were treated with itraconazole 200-400 mg per day and one case with terbinafine 250 mg per day. Among the eight cases treated wit itraconazole, case 3 and case 6 lost follow-up after prescribing the dose for half a month. The course of treatment ranged from 3 to 10 months in 7 cases. Case 7,case 8 and case 9 failed to consolidate treatment due to the interference of comorbidity. The totle rate of complete remission was 57.1% (4/7) in our study.

Discussion

Chromoblastomycosis in China is primarily caused by *C. carrionii* in the north and by *F. monophora* and *F.pedrosoi* in the southern and eastern parts of the country,[11] especially in Guangdong Province and Shandong Province of Mainland China.[8] *F.pedrosoi* and *C. carrionii* infections are normally observed in tropical and subtropical areas of endemicity around the world.[12] These are dematiaceous fungi living as saprophytes on plants or vegetable debris in the soil.[13] Zhejiang is located on the southeast coast of China and in the middle of subtropical zone, with monsoon humid climate. *C. carrionii* occurs in semiarid areas, whereas *Fonsecaea pedrosoi* is associated with humid climates.[12] In terms of geography and climate,Zhejiang is suitable for the survival of these dematiaceous fungi. Molecular phylogeny has facilitated the identification of cryptic species within the *F. pedrosoi* species complex. The isolates identified as *F.pedrosoi* morphologically now belong to *F. pedrosoi sensu strico*, *F. monophora,* [14] and *F. nubica*.[7] *F. pedrosoi* and *F. nubica* are strictly associated with chromoblastomycosis, whereas *F. monophora* is also involved in phaeohyphomycosis of brain and other organs.[7] Both *F. pedrosoi* and *F. monophora* are easily misidentified in clinical practice because both have similar morphological features. ITS region can confirm the species in most of the circumstances though it may have limitations. These limitations may be overcome by sequencing other loci and/or by other molecular methods. *CDC42*, *ACT1*, *BT2*, lactase (*Lac*),homogentisate (HmgA) and polyketide synthase (*PKS1*) (the first three genes were most common) combined with ITS for sequencing can increase the accuracy.[15] Based on our study, *F. monophora* is the dominant pathogen in CBM cases in Zhejiang. *F. monophora* isolates exhibited high genetic diversity.[16] So,long-term surveillance and molecular identification at the species level for all Fonsecaea isolates from CBM cases is necessary for epidemiology .

In agreement with previous literature, CBM cases are prevalent among males and farmers.Chromoblastomycosis is considered an occupational disease around the world,affecting farm laborers, gardeners, lumberjacks, vendors of farm products, and other workers exposed to contaminated soil and plant materials.[12] On rare occasions, wood fragments containing muriform cell-like structures were histopathologically observed in patients with CBM. [17,18] In our series,carpenter was the second

most common occupation. CBM is usually gradual and indolent in nature. The duration of disease at the time of presentation varied from 20 days to 35 years. [3] Most of the studied cases were chronic, with a mean duration of 6,5 years. The lower limbs are the area of the body that would be most likely to be in contact with material contaminated by fungi.[19] In most series, there has been a predilection for the lower legs,[2,3,19,20] However, in Australia, the upper limbs were more affected by CBM, [21] as in our cohort,predominantly the hand, wrist and forearm. The difference might be attributable to differences in economic structures among these countries. Rural workers in eastern China tend to wear protective clothing and shoes that covers the legs but not the hands and forearms. The most frequent clinical aspect of CBM are different in various reports, such as verrucous lesions,[19,22,23,22] nodular lesions,[24,22] cicatricial lesions,[2] and plaquelike lesions[3] were the predominance,respectively. Plaque was the most common in our study,followed by verrucous. Consistent with the results from previous reports, [19,20] any history of trauma was recalled only in 45.5% of the cases.This may be related to insensitivity to trauma when engaged in agricultural activities. Except for three cases infection via penetration of wood splinters,two cases had history of insect bite which may be a route of inoculation of soil or vegetative matter contaminated by dematiaceous fungi. Recently, CBM has been increasingly reported in immunocompromised individuals. In Taiwan, diabetes is the most common comorbidity.[16] In our series,diabetes and kidney trouble were the predisposing conditions.

The diagnostic characteristic is the presence of brown muriform cells in the infected tissue, which can be demonstrated in potassium hydroxide (KOH) mount and hematoxylin and eosin staining.[25] The sensitivity of direct examination ranges from 90 to 100%. This method is fast, easy, and inexpensive.[12] In the current study, microscopy showed muriform cells with or without germinating hyphae in the lesion scales of all patients. The tissular response is not specific in CBM specimens, and it may be similar to the tissue reactions observed for most implantation mycoses.[12] Pires et al.found two main types of granulomatous tissue reaction: suppurative granuloma with abundant fungal cells, mostly from verrucous lesions, and tuberculoid granuloma, with few parasites, from plaque and atrophic lesions.[26] The histopathology showed that muriform cells were present in 45.4% of our cases, while it was noted in 100% of cases reported by Mead and Ridley in 1957, [27] and in 12 of the 13 cases reported by Leslie and Beardmore in 1979.[28] CBM lesions are chronic, indolent, and clinically polymorphic. CBM can mimic a wide spectrum of diseases with infectious and noninfectious causes,[12] such as cutaneous tuberculosis, leprosy, leshmaniasis, sporotrichosis, mycetoma, psoriasis, and malignancies such as verrucous carcinoma and cutaneous lymphoma.[29] Our study confirmed that 72.7% (7/11) had been misdiagnosed. This suggest the importance to Improve the diagnostic ability of clinicians in rural areas.

CBM is difficult to treat and associated with low cure rates and high relapse rates, especially in chronic and extensive cases. Treatment choice and results depend on the etiological agent, size and extent of the lesions, topography, and presence of complications.[30] Patients showing severe and advanced clinical forms of disease require a long duration of continuous systemic antifungal treatment.[31,32] Itraconazole is the most common choice of first-line agents.[16] Itraconazole (200-400 mg/day) was the most common choice of first-line agent in our practice.The complete response rate was 57.1% (4/7) in our study. Among the 4 patients with a complete response,all patients were treated with systemic agents,with duration of treatment varied from 4-10 months.CBM mainly affects agricultural workers and rural population.These patients are often poor and unable to bear the burden of long-term treatment. In our series,1 case gave up treatment , 2 cases could not adhere to the treatment regimen and were lost of follow-up. The burden and medical impact of this implantation mycosis are

certainly underestimated. The use of protective equipment such as gloves, shoes, and adequate clothes may be a key point for reducing the risk of infection.[12]

In conclusion,CBM is a neglected fungal disease that mainly affects low-income agricultural workers and rural population. The diagnosis of CBM requires laboratory confirmation by direct mycological examination and/or histopathology. Visualization of muriform cells in clinical samples is a cornerstone of the diagnosis of this disease. In addition to the disease itself, treatment choice and results also depends on the patient's economic situation and comorbidity. Early detection and treatment is the key to cure the disease. Except for morphological identification, molecular analysis is important,especially in *Fonsecaea ~~spp~~. F. monophora* is identified as the major pathogen and involvement of the hands and forearms is more usual in Zhejiang.

References

1. McGinnis MR. Chromoblastomycosis and phaeohyphomycosis: new concepts, diagnosis and mycology. *J Am Acad Dermatol.* 8 (1), 1-16 (1983).

2.Fandresena Arilala Sendrasoa, Naina Harinjara Razanakoto, Mendrika Fifaliana Rakotoarisaona, Malalaniaina Andrianarison, Onivola Raharolahy, Tahinamandranto Rasamoelina, etal.Clinical aspects of previously treated chromoblastomycosis: A case series from Madagascar. *International journal of infectious diseases* : *IJID* : official publication of the International Society for Infectious Diseases. 101,228-232 (2020).

3.Reshu Agarwal, Gagandeep Singh, Arnab Ghosh, Kaushal Kumar Verma, Mragnayani Pandey, Immaculata Xess. Chromoblastomycosis in India: Review of 169 cases. *PLoS neglected tropical diseases.* 11(8):e0005534 (2017).

4.Arango M, Jaramillo C, Corte´s A, Restrepo A. Auricular chromoblastomycosis caused by Rhinocladiella aquaspersa. *Med Mycol* .36,43–5 (1998).

5.Son YM, Kang HK, Na SY, Lee HY, Baek JO, Lee JR, et al. Chromoblastomycosis caused by *Phialophora richardsiae*. *Ann Dermatol.* 22,362–6 (2010).

6. Najafzadeh MJ, Rezusta A, Cameo MI, Zubiri ML, Yus MC, Badali H, et al.Successful treatment of chromoblastomycosis of 36 years duration caused by *Fonsecaea monophora*. *Med Mycol* .48,390–3 (2010).

7. M J Najafzadeh, J Sun, V Vicente, L Xi, A H G Gerrits van den Ende, G S de Hoog. *Fonsecaea nubica sp. nov*, a new agent of human chromoblastomycosis revealed using molecular data.*Medical mycology* .48(6),800-6 (2010)

8.Sha Lu, Changming Lu, Junmin Zhang, Yongxuan Hu, Xiqing Li, Liyan Xi.Chromoblastomycosis in Mainland China: a systematic review on clinical characteristics.*Mycopathologia.* 175,5-6(2013)

9.Cindy Fransisca, Ya He, Zhiwen Chen, Hongfang Liu, Liyan Xi. Molecular identification of chromoblastomycosis clinical isolates in Guangdong. *Medical mycology.*55(8),896 (2017) .

10.Carrión AL. Chromoblastomycosis. *Ann N Y Acad Sci* 1950;50:1255–1282 https://doi.org/10.1111/j.1749-6632.1950.tb39826.x.

11.Liyan Xi, Jiufeng Sun, Changming Lu, Honfang Liu, Zhi Xie, Kazutaka Fukushima, et al. Molecular diversity of Fonsecaea (Chaetothyriales) causing chromoblastomycosis in southern China.*Medical mycology.* 47(1),27-33(2009).

12.Flavio Queiroz-Telles, Sybren de Hoog, Daniel Wagner C L Santos, Claudio Guedes Salgado, Vania Aparecida Vicente, Alexandro Bonifaz, et al. Chromoblastomycosis. *Clinical microbiology reviews.* 01;30(1),233-276 (2017).

13.M Pindycka-Piaszczyńska, P Krzyściak, M Piaszczyński, S Cieślik, K Januszewski, G

Izdebska-Straszak, et al. Chromoblastomycosis as an endemic disease in temperate Europe: first confirmed case and review of the literature. *European journal of clinical microbiology & infectious diseases* : official publication of the European Society of Clinical Microbiology. 33(3),391-8 (2014).
14.G S De Hoog, D Attili-Angelis, V A Vicente, A H G Gerrits Van Den Ende, F Queiroz-Telles.Molecular ecology and pathogenic potential of Fonsecaea species.*Medical mycology.* 42(5),405-16 (2004).
15.Zimeng You, Xiaoxiao Yang, Jianbin Yu, Jiangan Zhang, Yuping Ran. Chromoblastomycosis Caused by *Fonsecaea nubica*: First Report in Northern China and Literature Review.*Mycopathologia.* 184(1),97-105(2019).
16.Ching-Sheng Yang, Chun-Bing Chen, Yung-Yi Lee, Chih-Hsun Yang, Ya-Ching Chang, Wen-Hung Chung,et al.Chromoblastomycosis in Taiwan: A report of 30 cases and a review of the literature.*Medical mycology.* 01,56(4):395-405(2018).
17.Nuno Menezes, Paulo Varela, Antónia Furtado, Antonio Couceiro, Ismália Calheiros, Laura Rosado, et al. Chromoblastomycosis associated with *Fonsecaea pedrosoi* in a carpenter handling exotic woods. *Dermatology online journal.* 28,14(2):9 (2008).
18.A H Mehregan, E J Rudner. Implantation dermatosis. Wood splinter with fungus contamination. *Journal of cutaneous pathology.* 7(5),330-1 (1980).
19.Carla Andréa Avelar Pires, Marilia Brasil Xavier, Juarez Antônio Simões Quaresma, Geraldo Mariano Moraes de Macedo, Bruna Ranyelle de Marinho Sousa, Arival Cardoso de Brito. Clinical, epidemiological and mycological report on 65 patients from the Eastern Amazon region with chromoblastomycosis. *Anais brasileiros de dermatologia.* 87(4),555-60 (2012).
20.Gabriela Franco Marques, Paula Yoshiko Masuda, Juliana Martins Prazeres Sousa, Jaison Antônio Barreto, Patrick Alexander Wachholz. Clinical and demographic profile of chromoblastomycosis in a referral service in the midwest of São Paulo state (Brazil). *Anais brasileiros de dermatologia.* 90(1),140-2 (2015).
21.David Weedon, Mitchell van Deurse, Scott Allison, Cliff Rosendahl. Chromoblastomycosis in Australia: an historical perspective. *Pathology.* 08;45(5):489-91 (2013).
22.R Minotto, C D Bernardi, L F Mallmann, M I Edelweiss, M L Scroferneker. Chromoblastomycosis: a review of 100 cases in the state of Rio Grande do Sul, Brazil. *Journal of the American Academy of Dermatology.* 44(4):585-92 (2001).
23.A C Mello e Silva, A Serra Neto, C E Galvão, S G Marques, A C Saldanha, C M Pedroso e Silva, et al. *Fonsecaea pedrosoi*-caused chromoblastomycosis in the state of Maranhão. I. The clinical, epidemiological and evolutionary aspects. *Revista da Sociedade Brasileira de Medicina Tropical* . 25(1),37-44 (1992).
24.A Bonifaz, E Carrasco-Gerard, A Saúl. Chromoblastomycosis: clinical and mycologic experience of 51 cases.*Mycoses.*44(1-2),1-7 (2001).
25.M Ameen. Chromoblastomycosis: clinical presentation and management. *Clinical and experimental dermatology.*34(8),849-54 (2009).
26.Carla Avelar-Pires, Juarez Antonio Simoes-Quaresma, Geraldo Mariano Moraes-de Macedo, Marilia Brasil-Xavier, Arival Cardoso-de Brito. Revisiting the clinical and histopathological aspects of patients with chromoblastomycosis from the Brazilian Amazon region. *Archives of medical research.* 44(4),302-6 (2013).
27.M MEAD, M F RIDLEY. Sporotrichosis and chromoblastomycosis in Queensland.*The Medical journal of Australia.*16,44(7):192-7 (1957).

28.D F Leslie, G L Beardmore. Chromoblastomycosis in Queensland: a retrospective study of 13 cases at the Royal Brisbane Hospital. *The Australasian journal of dermatology.*20(1),23-30 (1979) .
29.Felix Boon Bin Yap.Chromoblastomycosis.*International journal of infectious diseases : IJID* : official publication of the International Society for Infectious Diseases.14(6),e543-4 (2010).
30.Arival Cardoso de Brito, Maraya de Jesus Semblano Bittencourt. Chromoblastomycosis: an etiological, epidemiological, clinical, diagnostic, and treatment update. *Anais brasileiros de dermatologia* .93(4),495-506(2018).
31.Liuqing Jiang, Wenyu Feng, Fang Li, Jingying Xu, Yanping Ma, Huiling Ma. Effect of One-methylcyclopropene (1-MCP) and chlorine dioxide (ClO_2) on preservation of green walnut fruit and kernel traits. *Journal of food science and technology.* 52(1),267-75 (2015).
32.Alexandro Bonifaz, Vanessa Paredes-Solís, Amado Saúl. Treating chromoblastomycosis with systemic antifungals. *Expert opinion on pharmacotherapy.*5(2),247-54 (2004).

TABLE 1. Clinical and fungal results of eleven cases of chromoblastomycosis

Case	Date	Age(years)/sex	Occupation	trauma	Clinical features/site	Duration(m)	Treatment regimen	outcome	Pathogen
**1**	2018.2	61/M	Farmer	No	Verrucous/right ankle	180	Lost to follow-up	No	*F. monophora*
**2**	2018,3	68/M	Carpenter	Yes	Cicatricial/left forearm	84	TBF (0.25 g/day) for 5 months	Cured	*C.carrionii*
**3**	2018,8	39/M	Farmer	No	Verrucous/buttocks	240	Lost to follow-up	No	*F. monophora*
**4**	2019.1	56/M	gardener	Yes	Plaque/left forearm	12	ITZ (0.2 g/day) for 4 months	Cured	*F. monophora*
**5**	2019.7	62/M	Carpenter	Yes	Plaque/trunk	168	ITZ (0.2-0.4	Cured	*F. monophora*

							g/day) for 10 months		
**6**	2019,10	71/F	Farmer	No	Plaque/right leg	18	Lost to follow-up	No	*F. monophora*
**7**	2020.3	83/M	gardener	No	Plaque/left wrist	5	ITZ (0.2 g/day) for 3 months	Partial cured	*F. monophora*
**8**	2020.5	70/F	Farmer	No	Plaque/right forearm	120	ITZ (0.4 g/day) for 3 months	Partial cured	*F. monophora*
**9**	2021.3	57/M	Farmer	No	Plaque/left wrist	18	ITZ (0.2 g/day) for 5 months	Partial cured	*F. monophora*
**10**	2021.5	68/M	Farmer	Yes	Nodular/left forearm	3	Lost to follow-up	No	*F. monophora*
**11**	2021.8	57/M	Carpenter	Yes	Plaque/right wrist	8	ITZ (0.4 g/day) for 4 months	Cured	*F. monophora*

F, female; M, male; ITZ, itraconazole; TBF, terbinafine;m,month

Fig.1.Clinical manifestations of chromoblastomycosis (CBM) cases. Verrucous lesions on the ankle (A) and the buttocks (B). Erythematous plaque-type was the most common presentation in our study (C). Nodular type (D) and cicatricial type of lesion with black crust (E) on the forearm.

Fig.2. Mycological features of CBM. Pigmented muriform cell with obvious septation (A) and brown muriform cell with germinated hyphae (B) (10% KOH,×400). Mixed granuloma with sclerotic body (PAS,×400).Slide culture on potato dextrose agar at 25°C on day 8 of *C.carrionii* (C) and *F. monophora*(E) (×400). *F. monophora* colonies on SDA at 25°C on day 7 (F).

Click here to access/download;Figure;Fig.1.jpg

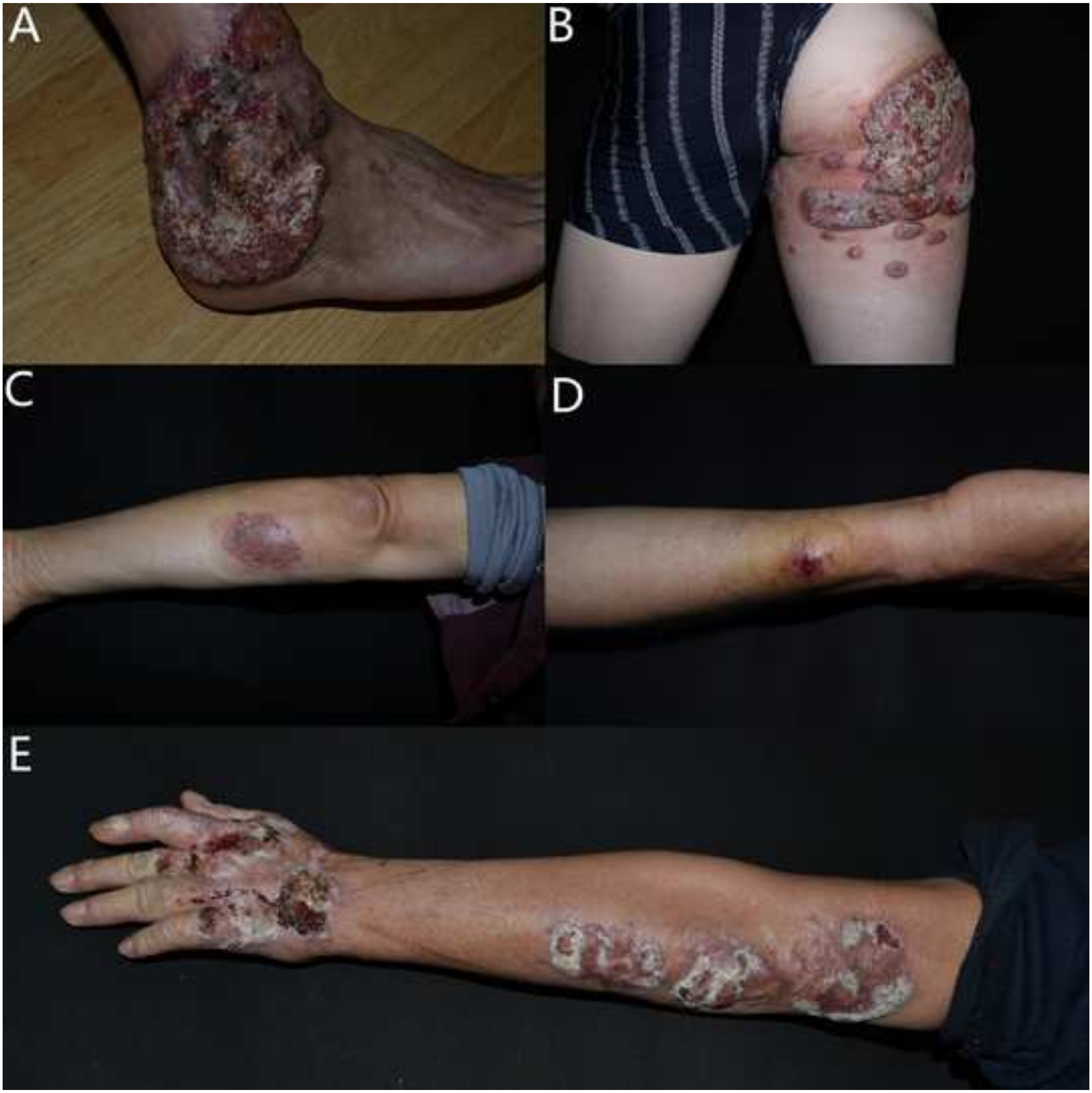

Figure

Click here to access/download;Figure;Fig.2.jpg

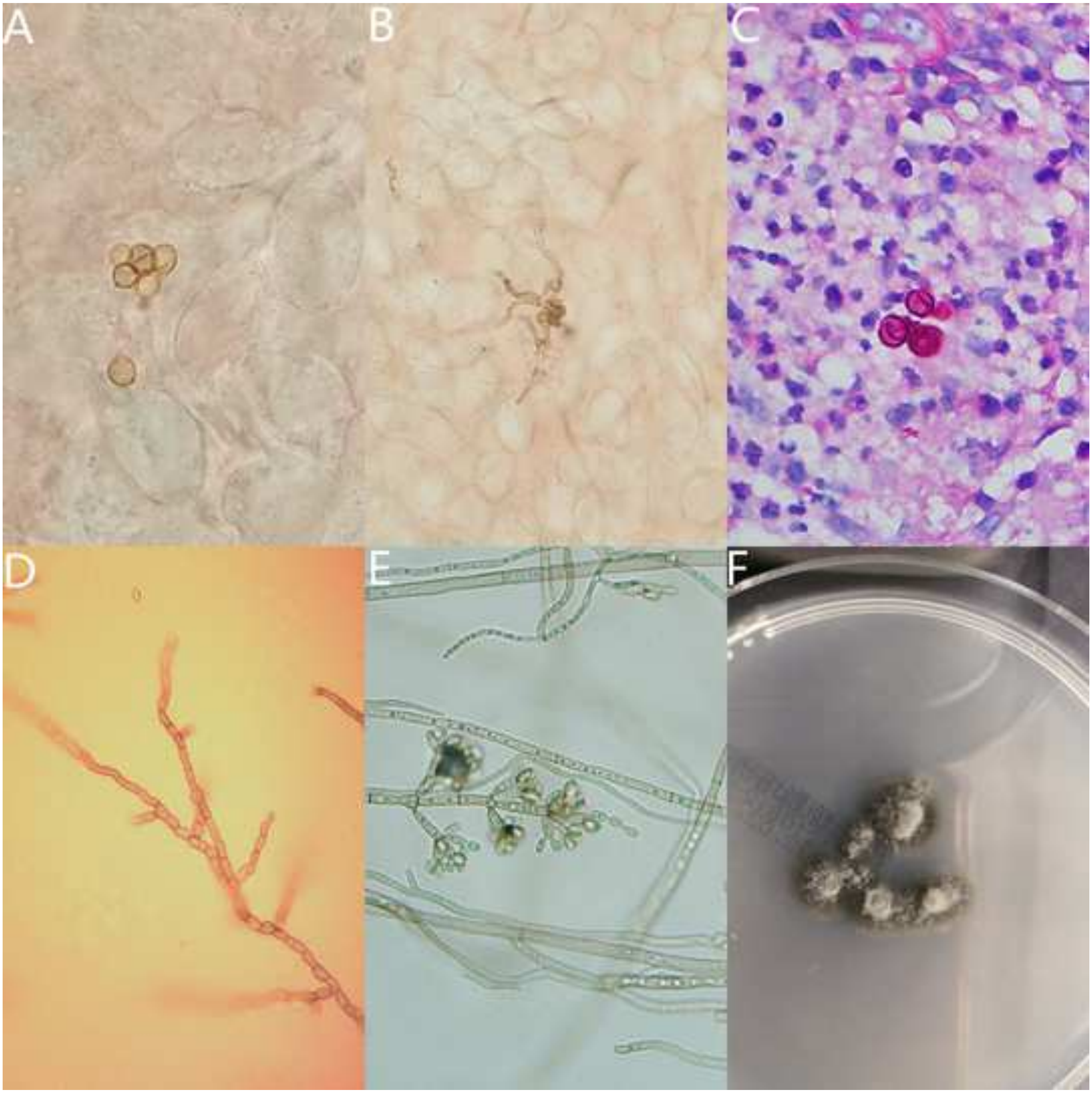

Table Click here to access/download;Table;TABLE 1.docx

TABLE 1. Clinical and fungal results of eleven cases of chromoblastomycosis

Case	Date	Age(years)/sex	Occupation	trauma	Clinical features/site	Duration(m)	Treatment regimen	outcome	Pathogen
**1**	2018.2	61/M	Farmer	No	Verrucous/right ankle	180	Lost to follow-up	No	*F. monophora*
**2**	2018,3	68/M	Carpenter	Yes	Cicatricial/left forearm	84	TBF (0.25 g/day) for 5 months	Cured	*C.carrionii*
**3**	2018,8	39/M	Farmer	No	Verrucous/buttocks	240	Lost to follow-up	No	*F. monophora*
**4**	2019.1	56/M	gardener	Yes	Plaque/left forearm	12	ITZ (0.2 g/day) for 4 months	Cured	*F. monophora*
**5**	2019.7	62/M	Carpenter	Yes	Plaque/trunk	168	ITZ (0.2-0.4 g/day) for 10 months	Cured	*F. monophora*
**6**	2019,10	71/F	Farmer	No	Plaque/right leg	18	Lost to follow-up	No	*F. monophora*
**7**	2020.3	83/M	gardener	No	Plaque/left wrist	5	ITZ (0.2 g/day) for 3 months	Partial cured	*F. monophora*
**8**	2020.5	70/F	Farmer	No	Plaque/right forearm	120	ITZ (0.4 g/day) for 3 months	Partial cured	*F. monophora*
**9**	2021.3	57/M	Farmer	No	Plaque/left wrist	18	ITZ (0.2 g/day) for 5 months	Partial cured	*F. monophora*
**10**	2021.5	68/M	Farmer	Yes	Nodular/left forearm	3	Lost to follow-up	No	*F. monophora*
**11**	2021.8	57/M	Carpenter	Yes	Plaque/right wrist	8	ITZ (0.4 g/day) for 4 months	Cured	*F. monophora*

F, female; M, male; ITZ, itraconazole; TBF, terbinafine;m,month
