# Supplementary material for: Chromoblastomycosis: A case series from Eastern China
Source: PLoS Negl Trop Dis. 2022 Sep 26;16(9):e0010800. doi: 10.1371/journal.pntd.0010800 (PMC9536625; doi:10.1371/journal.pntd.0010800)
Supplement: Attachment — Submitted filename: Chromoblastomycosis in Eastern China.docx [file pntd.0010800.s001.docx]

Dear authors your manuscript is interesting and describes chromoblastomycosis in the Eastern part of China.

I have a few suggestions or comments:

Please use “melanized fungi”, instead of “dematiaceous fungi”

In this phrase, “Diagnosis of these cases was made by clinical observation, mycological examination and histopathology of biopsies collected from scrapings of skin lesions”.

Please include that “culture” was also made in your patients.

Please use “living as saprobes” instead of “living as saprophytes”

Authors, why do you say that “Treatment choice and results depend on the etiological agent”?

There is no standardization for in vitro susceptibility tests for the melanized fungi, including *Fosecaea spp*. Itraconazole is the most experimented drug in patients with mild, moderate and severe forms pf chromoblastomycosis. According to the literature, the refractoriness of some patients is due to severity of the clinical form, local fibrosis and chronic secondary bacterial infection.

You used Carrion’s clinical classification for your patient series description. Why don’t you also grade the patients lesions according to their severity: mild, moderate and severe? There are robust publications showing that severity criteria may help clinician to better plan the therapy, in terms of antifungal dose and duration of treatment.
