# Supplementary material for: Chromoblastomycosis: A case series from Eastern China
Source: PLoS Negl Trop Dis. 2022 Sep 26;16(9):e0010800. doi: 10.1371/journal.pntd.0010800 (PMC9536625; doi:10.1371/journal.pntd.0010800)
Supplement: Attachment — Submitted filename: response.docx [file pntd.0010800.s003.docx]

Dear Editors：We have substantially revised our manuscript after reading the comments provided by four reviewers. We employed an English-language editing service （EditSprings） to polish our wording. We would like to thank you for the positive and constructive comments and suggestions. In order to check the follow-up status of patients, we followed up each patient by telephone, some patients uploaded photos after treatment to us through the Internet. So, some data is different from the first draft. Misdiagnosis was emphasised in the abstract and discussion of manuscript. other importmant cutaneous and subcutaneous infectious diseases alongside chromoblastomycosis were listed in the results. Thanks!

Answers to reviewers (Methods):

Reviewer #1: Please explain in more detail what is meant by a consultation campaign.were there regional organised clinics specifically seeking cases of chromoblastomycosis or skin disease in general. We need to know a bit more about this recruitment method. Was the mycological work, including direct microscopy carried out in a single laboratory ?

Answers: Thank you very much for your professional review of our manuscript, we benefit greatly by these suggestions. We are sorry, the phrase” consultation campaign”may be inaccurate. In the daily diagnosis and treatment activities of our department, according to the procedure, all cases with suspected importmant cutaneous and subcutaneous infectious diseases will be first arranged to come to the medical mycology laboratory for further evaluation and examination. So, this is a routine process. Epidemiological survey, mycological examination, including microscopic examination and culture, were carried out in the single laboratory. We have described each of these procedures in more detail in the methods section.

Reviewer #2: Suggestions and or comments were attached

Answers: The text has been modified accordingly. Thanks!

Reviewer #3: The authors retrospectively reviewed 11 cases of CBM in one center of Eastern China in three years.

Answers: Thanks!

Reviewer #4: The objective can be more clearly stated. It is a prospective study but in the introduction, it stated that "In order to evaluate the clinical characteristics of CBM in Zhejiang,eastern China,we reviewed 11 cases..," which may imply that it was a retrospective study instead.

In page 2, paragraph 1, the author stated: "Patients were recruited between January 2018 and December 2021 and during consultation campaigns conducted in Zhejiang Province.." What was meant by consultation campaigns? Were these "information campaigns" conducted in other clinics so that these kinds of cases were referred to the Hangzhou Third people's Hospital? It may good to describe further the "consultation campaign" and referral system followed since this would give readers an idea on the actual catchment area of the facility. Is Hangzhou Third people's Hospital a specialty hospital? Since it is a case series, conducted prospectively, it would have been better if pictures of individual cases were taken, and sizes of lesions were recorded. It would be interesting to note if cases with smaller lesion sizes responded to therapy faster and more completely, compared to those with larger and more disseminated lesions. Was surgery an option in any of the cases? If not, why not?

Answers: Thank you for your valuable suggestions! We are sorry for the confused expression. As mentioned above, this is a routine process in the daily diagnosis and treatment activities of our department. Since Xiujiao Xia initiated this clinical study in 2018, this process has become a conventional model. The description of the operation process in the introduction and methods has been adjusted. Pictures of individual cases had been taken, lesions were graded mainly based on the size of skin lesions. Surgery is an option in CBM cases, especially in lesion with small size, we will cooperate with cutaneous surgery in the future. Thanks!

Answers to reviewers (Results):

Reviewer #1: Please explain the following phrase - consolidate treatment due to the interference of comorbidity. Kidney trouble – better chronic renal disease.

Answers: The two patients with respectively lupus nephropathy and nephrotic syndrome have used immunosuppressants for a long time, due to abnormal renal function during the treatment of CBM, the treatment was forced to be interrupted. The expression has been adjusted. Thanks!

Reviewer #2: The use of lesions severity graduation would be this article more interesting for clinicians.

Answers: Thank you for your suggestion, we have added this part to the text .

Reviewer #3: The study indicated that the most common pathogenic fungus was F.monophora.

Answers: Yes, in recent years, every important pathogen has been molecular identified.

Reviewer #4: Figure 1 clinical photos are not clear. It would be good to indicate which particular cases these were. Since it is a case series, it would be good to include before and after treatment pictures as well.

Answers: Thank you for your comment. Figure 1A looks very dark, we chosed a brighter one. We also processed the rest of the pictures and added some photos of post treatment.

Answers to reviewers (Conclusions):

Reviewer #1: The authors argue for improving diagnostic ability in rural areas to reduce the risk of misdiagnosis. It would, be useful here to discuss possible methods of doing this. Please mention other forms of treatment such as oral terbinafine or heat therapy. It would be useful in the discussion to on the distribution and case load of chromoblastomycosis in China. How common is it ? Are there any explanations for the differ ent distribution of organisms ? Why is F monophora a dominant cause in China ? Is this a true geographic variation or is this because without molecular tools most such strains have been misidentified as F pedrosoi in the past ?

Answers: Thank you for your suggestion, we have added this part to the text. Direct microscopic examination is indeed a simple and effective method. We also expressed other suggestions in the manuscript. Thank you very much!

Reviewer #2: Suggestions and or comments were attached

Answers: The text has been modified accordingly. Thanks!

Reviewer #3: The authors should indicate the limitations of this case series, such as the limited number of the patients, single center study, etc.

Answers: Thanks! we have added this part in the end of the text.

Reviewer #4: The conclusions can be improved to refer more to the cases seen in the facility.

Answers: Thanks! We have perfected this part as much as possible. Thanks again.

Answers to reviewers (Editorial and Data Presentation Modifications):

Reviewer #1: There are some changes needed to the English. Please mention that chromoblastomycosis is formally designated by WHO as an NTD

Answers: We employed an English-language editing service EditSprings to polish our wording. We have quoted this statement：“Chromoblastomycosis (CBM) is a neglected tropical disease (NTD).^12^ ” Thank you for your suggestion！

Reviewer #2: (No Response)

Answers: Thanks!

Reviewer #3: None

Answers: Thanks!

Reviewer #4: Page 4, paragraph 1: associated condition, not predisposing

Page 3, paragraph 1: 8 cases were misdiagnosed; Page 4, paragraph 2: 7 cases were diagnosed. Please clarify.

Answers: Thank you for your correction. We are sorry for our carelessness.

Answers to reviewers (Summary and General Comments):

Reviewer #1: (No Response)

Answers: Thanks!

Reviewer #2: Suggestions and or comments were attached

Answers: The text has been modified accordingly. Thanks!

Reviewer #3: The novelty of this study is relatively low.

Answers: Thanks!

Reviewer #4: 1. There may be a need to edit the article for it to be publishable.

2. These are the points that I think are good take-aways from this article

a. All cases were initially evaluated in other clinics before the information campaign and 8 were misdiagnosed. This highlights the importance of educating primary physicians or those who usually initially manage these cases, on recognizing chromoblastomycosis and other skin neglected tropical diseases. b. All 11 cases were diagnosed with chromoblastomycosis after doing a simple, easy, non-invasive, inexpensive, out-patient procedure, the KOH smear. This illustrates that KOH smear may even be more sensitive in catching these cases compared to punch biopsy.

Answers: Thank you for your insight！We try to integrate your suggestions into the text as much as possible.
