# Supplementary material for: Chromoblastomycosis: A case series from Eastern China
Source: PLoS Negl Trop Dis. 2022 Sep 26;16(9):e0010800. doi: 10.1371/journal.pntd.0010800 (PMC9536625; doi:10.1371/journal.pntd.0010800)
Supplement: Attachment — Submitted filename: response(R2).docx [file pntd.0010800.s004.docx]

Dear Editors：We are pleased to note the favorable comments of reviewers. Thanks!

Answers to reviewers:

1:Please remove the term dematiaceous and replace with brown pigmented - throughout the manuscript, including the abstract. There are still some errors of english, which should be improved before final publication. For example 'histopathology, not histopathology of biopsies'

In the abstract just state how many males, not a ratio.

Answers: We are seding the revised manuscript according to the comments of the reviewers. Revised portion are marked in red. Thank you very much!

2: The authors have added helpful information on the other similar presentations. Do they have any sense of trends over time? Is CBM diminishing in frequency, or static? And how does it compare to sporotrichosis, for example.

Answers: Thank you for your valuable suggestions! We made a graph to reflect the trend of these diseases. Thanks!

Best wishes!
